# Phosphorus Necrosis

**Published:** 1862-10

**Authors:** 


Phosphorus Necrosis.—In a recent clinical lecture by Mr. Paget, in speaking of a case of phosphorus necrosis, he remarked as follows:

In this case there was a special interest in the fact that it was an exception to the almost absolute rule that the disease only occurred in lucifer-match makers. Lucifer-match makers were hard worked, and were kept in a bad atmosphere, and to this, and not to the phosphorus, some had attributed the disease. Any one long enough exposed to the fumes of phosphorus suffered from necrosis of one or both jaws, and the majority employed in the manufactories suffered at one time or another from this disease. But there were certain conditions which were necessary before a person became liable to suffer. So long as the mucous membrane was intact, and the teeth sound, there was no mischief. This had been carefully ascertained, and was quite certain. In one of the large German manufactories it had been found that if the workmen had broken or decayed teeth, gumboils, or anything which bared the periosteum, they became liable to suffer, and this was confirmed by experiments on animals. It was found that if healthy rabbits were kept in a chamber into which the phosphorus fumes were admitted, no disease of the jaws followed. If, however, their teeth were broken, or if in any way the periosteum were exposed, then they began to suffer. Thus, then, it may be accepted as a well ascertained fact, that unless the phosphorus fumes come in contact with the periosteum, the disease is not produced.

The manner in which the fumes produce necrosis is also singular, unlike other caustics, as, for instance, nitric acid. It does not act merely locally, destroying what it touches, but it seems to affect more or less the neighboring bone,—part of it, or in time the whole of it. There is no other substance which, when locally applied, seems to produce results over so large a space. Not unfrequently it would thus destroy the whole bone attacked, beginning first at a single point of bared periosteum, as the socket of a tooth after extraction. In this peculiarity of action, it appears to resemble less a mineral than an organic poison.

So far as has been at present ascertained, no bones except those of the upper and lower jaw have been attacked; altho' of course, now and then, there must have been, as from accidental strumous disease, exposure of other bones to the fumes. The contiguous nasal bones never suffered, at least not primarily, but sometimes as a sequence to disease of the jaw bones. This curious choosing of certain structures shows that these bones have distinctive peculiarities, for which, by comparison of their texture with other bones, we can not yet account. It was just as difficult to tell why mercury should affect the jaws more than other bones. It is the more difficult to explain why these bones should suffer from mercury and phosphorous acid, as they are not liable to be affected by organic diseases, as syphilis or gout.

In reference to the way in which the necrosis was brought about, Mr. Paget said that inflammation of the periosteum was the first step. He showed a specimen in which new bone had been deposited at many points, as a result of this process. This was a constant first effect, and new bone was always to be found, more in the lower than in the upper jaw. In a few —very few—cases the disease would stop here. Generally, however, the result was necrosis of the whole of the affected bone, and that, too, of the new bone as well as the old.

In the case of removal of the greater part of the lower jaw (the first case related), the periosteum of the remaining part of the bone was inflamed, and probably to some extent the necrosed bone might be replaced by new bone.

In a case of phosphorus necrosis, it was difficult to say, when once begun, how it would end. In some a limited part of the bone would separate, but in others the whole substance of the maxilla would become necrosed. Mr. Paget had observed that the patients were much disordered in health, but he would not venture to say that there was a special cachexia in this disease. There was, however, much more cachexia than was usual in local disease to that extent. The patients were peculiarly pale, sodden-looking, and exceedingly feeble. They were not thin, but looked as if the blood were deficiently red. There was generally also some bronchitis. It was a matter of inquiry whether the phosphorous acid acted as a poison in the blood—the necrosis being the local manifestation of it.

An important question was, Could the disease, by proper care, be entirely prevented ? In some conditions of perfect ventilation, and great care as to the condition of the mouth in those exposed, no doubt it could. Since 1847, in the manufactory at Nuremberg, only one case had occurred in fourteen years. This improvement was due to perfect ventilation, great cleanliness, and inspection of the teeth. It is while dipping the matches that exposure to the fume occurs. It is necessary that the fumes should, as much as possible, be blown away from the workmen. No one with broken teeth, gumboils, etc., should be allowed to work at dipping the matches.

The allotropic phosphorus does not fume, and thus, so far as the health of the workmen is concerned, is better. It is, however, more expensive, and more liable to take fire. It is to be regretted, Mr. Paget said, that similar regulations to those in the German manufactories are not adopted in England.

In treating this disease when established, we ought of course to advise removal from the influence of the fumes, fresh air and good diet, and thus hope to limit the necrosis. Is it advisable, when the necrosis is limited, to cut it out? Mr. Paget believed it was ; but it was necessary to be cautious in taking for granted that the necrosed part was the only part diseased. There might be periostitis, in the earlier stages of the disease, beyond, so that by the removal of the necrosed bone it is not clear that we should relieve.

After removal in these cases, the repair is very remarkable. In the case of the boy in Darker Ward, there was a very large quantity of new bone in the position of the ramus and body. Mr. Paget said that he had seen no case in this disease in which death had occurred, but it would sometimes prove fatal by exhaustion and the supervention of phthisis. —*Med. Times and Gazette.*

